# Functionally distinct regions of the locus *Leishmania major response 15* control IgE or IFNγ level in addition to skin lesions

**DOI:** 10.3389/fimmu.2023.1145269

**Published:** 2023-08-03

**Authors:** Imtissal Krayem, Yahya Sohrabi, Helena Havelková, Elena S. Gusareva, Hynek Strnad, Marie Čepičková, Valeryia Volkova, Iryna Kurey, Jarmila Vojtíšková, Milena Svobodová, Peter Demant, Marie Lipoldová

**Affiliations:** ^1^ Laboratory of Molecular and Cellular Immunology, Institute of Molecular Genetics, Czech Academy of Sciences, Prague, Czechia; ^2^ Department of Medical Genetics, Third Faculty of Medicine, Charles University, Prague, Czechia; ^3^ Department of Cardiology I-Coronary and Peripheral Vascular Disease, Heart Failure, University Hospital Münster, Westfälische Wilhelms-Universität, Münster, Germany; ^4^ Department of Genomics and Bioinformatics, Institute of Molecular Genetics of The Czech Academy of Sciences, Prague, Czechia; ^5^ Department of Parasitology, Faculty of Science, Charles University, Prague, Czechia; ^6^ Department of Molecular and Cellular Biology, Roswell Park Comprehensive Cancer Center, Buffalo, NY, United States

**Keywords:** *Leishmania major*, susceptibility to infection, quantitative trait locus, advanced intercross line, recombinant mapping, bioinformatics analysis, fine mapping, functional heterogeneity

## Abstract

Leishmaniasis, a disease caused by parasites of *Leishmania* spp., endangers more than 1 billion people living in endemic countries and has three clinical forms: cutaneous, mucocutaneous, and visceral. Understanding of individual differences in susceptibility to infection and heterogeneity of its pathology is largely lacking. Different mouse strains show a broad and heterogeneous range of disease manifestations such as skin lesions, splenomegaly, hepatomegaly, and increased serum levels of immunoglobulin E and several cytokines. Genome-wide mapping of these strain differences detected more than 30 quantitative trait loci (QTLs) that control the response to *Leishmania major*. Some control different combinations of disease manifestations, but the nature of this heterogeneity is not yet clear. In this study, we analyzed the *L. major response* locus *Lmr15* originally mapped in the strain CcS-9 which carries 12.5% of the genome of the resistant strain STS on the genetic background of the susceptible strain BALB/c. For this analysis, we used the advanced intercross line K3FV between the strains BALB/c and STS. We confirmed the previously detected loci *Lmr15*, *Lmr18*, *Lmr24*, and *Lmr27* and performed genetic dissection of the effects of *Lmr15* on chromosome 11. We prepared the interval-specific recombinant strains 6232HS1 and 6229FUD, carrying two STS-derived segments comprising the peak linkage of *Lmr15* whose lengths were 6.32 and 17.4 Mbp, respectively, and analyzed their response to *L. major* infection. These experiments revealed at least two linked but functionally distinct chromosomal regions controlling IFNγ response and IgE response, respectively, in addition to the control of skin lesions. Bioinformatics and expression analysis identified the potential candidate gene *Top3a*. This finding further clarifies the genetic organization of factors relevant to understanding the differences in the individual risk of disease.

## Introduction

More than 1 billion people living in endemic countries (1–3) are endangered by leishmaniasis, a disease with no reliable vaccine to prevent it in humans. Moreover, treatment of leishmaniasis has serious side effects (4, 5).

The disease is caused by kinetoplastid parasites of the genus *Leishmania* that are transmitted to mammalian hosts by a bite of the vector, phlebotomine sand flies (Diptera). In the infected mammalian organism, *Leishmania* parasites invade “professional phagocytes,” including monocytes, macrophages, and neutrophils, and can also reside in dendritic cells and many other cell types such as fibroblasts (6) and adipocytes (7). The disease has three main forms: cutaneous, mucocutaneous, and visceral. The clinical form and the susceptibility to leishmaniasis depend on parasite species, pathogen transmission vector, immune status, nutrition, age, sex, microbiome and genotype of the host, and also on multiple environmental and social factors and co-infections (8–13).

These multiple factors are difficult to control in the analysis of susceptibility to leishmaniasis in humans and are easier to control in animal models, even if they cannot cover all the variabilities of human leishmaniasis. A broad range of disease manifestations such as skin lesions, splenomegaly, hepatomegaly, parasite infiltration into the organs, eosinophil infiltration into the lymph nodes, and increased levels of immunoglobulin E and cytokines in the serum were described in different mouse strains (14–17) and animal models proved to be invaluable in revealing the mechanisms (18–22) and genetic architecture (8, 9, 13, 18) of response to leishmaniasis. In mouse, the most detailed information was obtained in the studies of infection with *L. major* (8, 9, 13). Genome-wide mapping detected more than 30 quantitative trait loci (QTLs), revealing the multigenic control of disease susceptibility and manifestations (23–25).

Some of these QTLs control different combinations of disease manifestations, but the nature of this heterogeneity is not yet clear. Moreover, the controlling genes are involved in one or more genetic interactions, functioning as a network (13, 25, 26). Although the system of recombinant congenic strains (RCS) allows by mapping in F_2_ hybrids to localize some QTLs to a short segment up to 1.78 Mb/<1 cM (*cora1*) (27), the majority of QTLs detected in RCS and other types of crosses are mapped to segments of 20 cM or more (23–25) that have to be further shortened to identify the controlling gene. Only one candidate gene *Fli1* controlling the susceptibility to *L. major* in mouse has been identified until now in a genome-wide search (28).

In this study, we analyzed four out of eight already mapped loci controlling the response to *L. major* in the RC strain CcS-9 (**Table 1**) (17, 29) using the advanced intercross line K3FV. The strongest linkage was observed to the *Lmr15* on chromosome 15. We prepared interval-specific strains covering the peak of this linkage. The analysis led to the confirmation, precise mapping, and identification of potential candidate genes in the locus *Lmr15*.

**Table 1:** Loci controlling response to *Leishmania major* in the strain CcS-9.

Chromosome	Locus	Controlled trait	Reference
**2**	*Lmr14*	Parasite load in the lymph nodes (males main; both sexes int. *Lmr25*, int. *Lmr24*); parasite load in the liver int. *Lmr27*; IL-13 in the serum; eosinophil infiltration into the lymph nodes	(17, 29)
**4**	*Lmr24*	Skin lesions; splenomegaly; parasite load in the lymph nodes—int. *Lmr14*; IL-4 and IFNγ in the serum; IL-10 in the serum—int. *Lmr4*	(29)
**5**	*Lmr25*	Parasite load in the lymph nodes—int. *Lmr14*; eosinophil infiltration into the lymph nodes; parasite load in the liver int. *Lmr27*	(17, 29)
**6**	*Lmr4*	Parasite load in the lymph nodes—males—int. *Lmr27*; IL-10 in the serum	(29)
**9**	*Lmr26*	Eosinophil infiltration into the lymph nodes int. *Lmr15*	(17)
**11**	*Lmr15*	Skin lesions (main and int. *Lmr18*); splenomegaly; hepatomegaly; parasite load in the lymph nodes (main, int. *Lmr27* in males); parasite load in the liver; eosinophil infiltration into the lymph nodes int. *Lmr26*; IL-4 and IgE in the serum	(17, 29)
**16**	*Lmr18*	Skin lesions int. *Lmr15*	(29)
**17**	*Lmr27*	Parasite load in the lymph nodes—males—main, int. *Lmr15*, int. *Lmr4*; parasite load in the liver—int. *Lmr14*	(29)

int., interaction.

main; main effect.

## Materials and methods

### Mice

We have used in these studies the genetic combinations of genomes of the strain BALB/c that is widely used in research and the strain STS that originated from Swiss albino mice in 1955 (30). The strain STS is resistant to infection with *L. major* (31), *Leishmania tropica* (16), and tick-borne encephalitis virus (32). STS is resistant to mammary tumor induction by hypophysial isografts (33) and highly susceptible to the induction of colon tumors by 1,2-dimethylhydrazine (34). STS thymocytes were more resistant to radiation-induced apoptosis than BALB/c thymocytes (35), whereas STS mice were more susceptible to radiation-induced apoptosis in the colon than BALB/c (36). The splenocytes of STS show a higher proliferative response to IL-2 than BALB/c (37) but a lower response to anti-CD3 (37) and ConA (27) than BALB/c. STS exhibited a higher proliferative response in the mixed lymphocyte culture than BALB/c when tested with cells from 11 other mouse strains with 10 MHC types (38).

#### Advanced intercross line

The advanced intercross line (AIL) K3FV was established from a susceptible strain BALB/c and a resistant strain STS. It contained STS-derived segments on chromosomes 1, 2, 4, 5, 7, 8, 10, 11, 16, 17, and 18. Chromosomal segments containing the *Lmr* loci detected in F_2_ hybrids between K3FV and BALB/c were typed using the following markers: D4Nds3, D4Mit108, D4Mit53, D4Mit139, D4Mit7, D4Mit152, D11Mit20, D11Mit139, D11Mit141, D11Mit274, D11Mit26, D11Mit242, D11Nds18, D11Mit37, D16Mit19, D16Mit94, D16Mit155, D17Mit66, D17Mit139, D17Mit20, D17Mit3, D17Mit120, D17Mit38, D17Mit72, and D17Mit129.

For the current analysis, we selected sublines that did not carry STS-derived segments in the *Lmr* loci on chromosomes 1, 2, 7, 8, 10, and 18. The F_4_ generation of the (STS×BALB/c) AIL mice with recombination in *Lmr24* (chromosome 4), *Lmr15* (chromosome 11), *Lmr18* (chromosome 16), and *Lmr27* (chromosome 17) regions was used in the present study. We used F_4_ (STS×BALB/c) AIL (K3FV) that was backcrossed to BALB/c mice once (N1—experiment 1) or twice (N2—experiment 2). The length of the obtained individual regions was approximately 5 cM.

F_2_ hybrids between BALB/c and K3FV (*n* = 138, 68 males and 70 females) were infected at the age of 8 to 14 weeks (the mean age is 11 weeks; the median age is 11 weeks) and characterized for the immunological and pathological changes after *L. major* infection. They were tested in two subsequent experimental groups (F_2_N1, *n* = 34—experiments 1; F_2_N2, *n* = 104—experiment 2). During the experiments, male and female mice were placed in separate rooms and males were caged individually.

#### Interval-specific congenic strains

The interval-specific congenic strains 6232HS1 and 6229FUD with recombinant haplotype in *Lmr15* were produced from the recombinant congenic strains CcS-4 and BALB/c using marker-assisted breeding (39). F_2_ mice from the cross between CcS-4 and BALB/c were genotyped, and mice that contained STS alleles at *Lmr15* and BALB/c alleles at the other STS-derived segments were backcrossed to BALB/c and genotyped again. This resulted in the establishment of the interval-specific strains 6232HS1 and 6229FUD, which carried STS-derived segments on chromosome 11 at the *Lmr15* region on the genetic background of BALB/c. Mice were cleaned by embryo transfer.

F_2_ hybrids between BALB/c and 6232HS1, 150 females, were infected at the age from 9 to 16 weeks (mean age = 12.8 weeks; median age = 12 weeks). Mice were tested in a single experimental group. The microsatellite marker D11Mit316 was used for typing of the *Lmr15* region.

F_2_ hybrids between BALB/c and 6229FUD, 150 females, were infected at the age from 8 to 12 weeks (mean age = 11 weeks; median age = 11.4 weeks). Mice were tested in a single experimental group. The microsatellite marker D11Mit242 was used for typing of the *Lmr15* region.

All experiments were approved by the Ethical Committee of the Institute of Molecular Genetics.

### Genotyping of AIL and interval-specific mice

DNA was isolated from the tails using a standard proteinase K procedure (40). Microsatellite and single nucleotide polymorphism (SNP) markers (Generi Biotech, Hradec Králové, Czech Republic) were genotyped as described elsewhere (41, 42). The products were electrophoresed in 3% agarose gel containing 80% of MetaPhor^®^ Agarose (Cambrex Bio Science Rockland, Inc., Rockland, ME, USA) and 20% of UltraPure™ Agarose (Invitrogen, Carlsbad, CA, USA) for 20 min to 2 h at 150 V.

### Parasites

*Leishmania major* LV 561 (MHOM/IL/67/LRC-L137 JERICHO II) was maintained in rump lesions of BALB/c females. Amastigotes were transformed to promastigotes using SNB-9 (43), and 10^7^ promastigotes from 6-day-old subculture 2 were inoculated in 50 μl of sterile saline s.c. into mouse rump (25). This procedure results in approximately 17% of metacyclic promastigotes in the inoculum (44).

### Disease phenotype

The size of the primary skin lesions was measured weekly using a Vernier caliper gauge. The mice were killed 8 weeks after infection, and body, spleen, and liver weights were recorded. The blood, spleen, skin, lymph nodes, and liver (in interval-specific strains only) were collected for further analysis.

### IgE and IFNγ levels

IgE and IFNγ levels in the serum were determined using the primary and secondary monoclonal antibodies (IgE: R35-72, R35-118; IFNγ: R4-6A2, XMG1.2) and standards from Pharmingen (San Diego, CA, USA) (purified mIgE: C38-2 and recombinant mouse IFNγ). The enzyme-linked immunosorbent assay (ELISA) was performed as recommended by Pharmingen. The IFNγ and IgE levels were estimated using the curve fitter program KIM-E.

### Measurement of parasite load in the organs

Total DNA was isolated from frozen lymph nodes and liver samples, and parasite load was measured using PCR-ELISA according to the previously published protocol (45). Briefly, total DNA was isolated using a standard proteinase K procedure (40). For the detection of the *Leishmania* parasite DNA in total DNA, PCR was performed using two primers: digoxigenin-labeled F 5′-ATT TTA CAC CAA CCC CCA GTT-3′ and biotin-labeled R 5′-GTG GGG GAG GGG CGT TCT-3′ (VBC Genomics Biosciences Research, Austria). The 120-bp fragment within the conserved region of the kinetoplast minicircle of the *Leishmania* parasite was amplified. In each PCR reaction, 50 ng of extracted total DNA was used. As a positive control, 20 ng of *L. major* DNA per reaction was amplified as the highest concentration of the standard. A 26-cycle PCR reaction was used for the quantification of parasites in the lymph nodes and liver. Parasite load was determined by measurement of the PCR product with the modified ELISA protocol (Pharmingen, San Diego, USA). The concentration of *Leishmania* DNA was measured using the ELISA Reader from Tecan with the curve fitter program KIM-E (Schoeller Pharma, Prague, Czech Republic) using least squares-based linear regression analysis (45, 46).

### RNA isolation and RT-PCR analysis

RNA was prepared by lysing skins and spleens stored at −80°C with the TRI reagent (Sigma-Aldrich, Missouri, United States) and analyzed as described in (47). One microgram of RNA was treated with DNase (Promega, Wisconsin, United States, M6101) and then reverse-transcribed using 100 units of M-MLV Reverse Transcriptase (Sigma, M1302) with 1×MLV reverse transcriptase buffer, 1.4 µM of random hexamers (Thermo Fisher, Massachusetts, United States, N8080127), 2.5 units of ribonuclease inhibitor (Thermo Fisher, 15518012), and 5 mM of each dNTP (Sigma, DNTP100) per sample to obtain cDNA. cDNA was then diluted five times and 3 µl was used for amplification by 45 cycles of PCR: 2 min denaturation at 95°C, 15 s denaturation at 95°C followed by 20 s annealing at 60°C and 30 s extension at 72°C with a single fluorescence acquisition point repeated 45 times, and a melt curve program of 55°C to 95°C with 0.5°C increment with continuous fluorescence acquisition using primers for the genes of interest and SYBR^®^ Green JumpStart™ Taq ReadyMix™ (Sigma-Aldrich, S4438) for quantification. GAPDH was used as an internal control. Reactions were performed in a 384-well plate in Roche light cycler LC480II (Roche Molecular Systems, Inc., Basel, Switzerland). Forward and reverse sequences of primers for the genes of interest were designed by QuantPrime (48) and purchased from Generi Biotech (Hradec Králové, Czech Republic). The sequences of the forward (F) and reverse (R) primers used were as follows: *Top3a_F*: GTGGCGAAGGCAAAGAAGTTGG; *Top3a_R*: TCTTCTTGCTGGGCCATCTCTG; *Aloxe3_F*: AGCCCGCCAAGAATGTTATCGC; *Aloxe3_R*: TCCTGAAAGCTGCTGACATCCAC; *Arhgap44_F*: TGACATGAGTGGCGCAGTGTTG; *Arhgap44_R*: GGGACATCAAAGTGGACGAGATCC; *Gapdh_F:* AACTTTGGCATTGTGGAAGG; *Gapdh_R*: GTCTTCTGGGTGGCAGTGAT.

### Detection of polymorphisms that change RNA stability and the functions of genes

We have sequenced the genomes of the strains BALB/c and STS using the next-generation sequencing (NGS) system HiSeq 2500 (Illumina, California, United States) (12× coverage) and analyzed them as described in (49, 50). In detail, NGS data were preprocessed using the software Trimmomatic (51), and overlapping pair reads were joined by the software Flash (52). Alignment-reference mouse sequence mm10 (build GRCm38) was performed using the Burrows–Wheeler Aligner (BWA) program (53). Mapped reads were sorted and indexed, and duplicated reads were marked. Local realignment around indels, base recalibration, and variant filtration were performed using the software Genome Analysis Toolkit (GATK) (54). The Integrated Genome Viewer (IGV) (55) was used for the visualization of results. Variant annotation and effect prediction was performed by the software SnpEff (56). Protein variation effect predictions were performed by the software Protein Variation Effect Analyzer (PROVEAN) (57). Analysis of conservation scores was performed using the ConSurf software (58–60).

### Statistical analysis

Peaks of linkage (association) for different parameters in the strain K3FV were estimated using an open-source PLINK program (https://zzz.bwh.harvard.edu/plink/plink2.shtml; http://pngu.mgh.harvard.edu/~purcell/plink/) by Shaun Purcell at the Center for Human Genetic Research, Massachusetts General Hospital, and the Broad Institute of Harvard & MIT (61).

Interval-specific congenic strains HS1 and FUD. The role of genetic factors in the control of skin and organ pathology, parasite load in the lymph nodes and liver, and also IgE or IFNγ level in the serum was examined with one-way analysis of variance followed by Bonferroni’s multiple comparison test using GraphPad Prism version 5.04. When necessary, the original values of an analyzed parameter were transformed for normalization of the distribution as described in the legends to the figures.

## Results

### Analysis using AIL K3FV confirmed the presence of the previously detected *Lmr* loci

**Figure 1:**
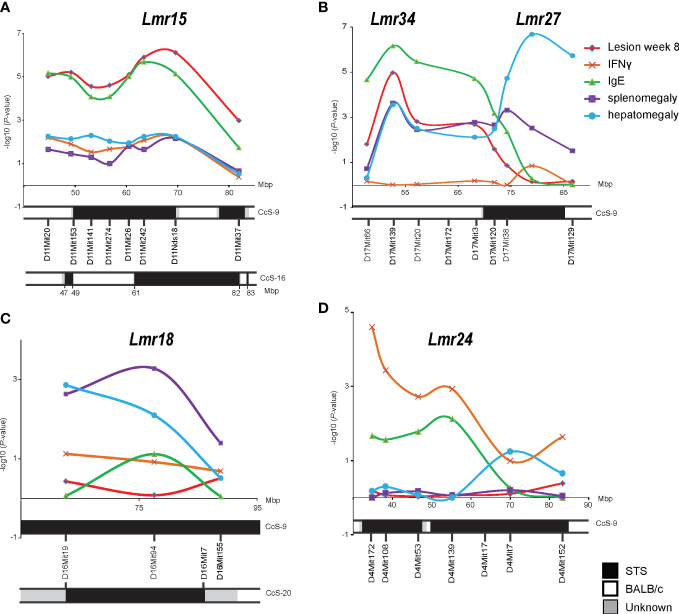
Mapping in the advanced intercross line K3FV. *P*-values organized by marker chromosomal locations on chromosome 11 **(A)**, chromosome 17 **(B)**, chromosome 16 **(C)**, and chromosome 4 **(D)**. IFNγ – IFNγ level in serum 8 weeks after infection; IgE - IgE level in serum 8 weeks after infection.

We have tested the association of skin lesions, splenomegaly, hepatomegaly, and IFNγ and IgE levels in the serum to the *Lmr* loci on chromosomes 4, 11, 16, and 17 using AIL K3FV (**Figure 1**).

The strongest linkage was detected to *Lmr15* on chromosome 11, and the peak of linkage to lesion size was observed between D11Mit242 and D11Nds8, whereas the peak of linkage to IgE level was found between D11Mit26 and D11Nds18 (**Figure 1A**).

We have detected the linkage of skin lesions, splenomegaly, and IgE level to *Lmr27* (**Figure 1B**). However, the AIL K3FV covers not only *Lmr27* on the distal part of chromosome 17 but also more proximal segment between 45 and 67 Mbp that is absent in CcS-9. We have detected a linkage to this segment that was associated with the controls of skin lesions, splenomegaly, hepatomegaly, and IgE level in the serum; a peak of linkage was observed around D17Mit139 (52.9 Mbp) (**Figure 1B**). This newly detected locus was named *Lmr34*.

Linkage to splenomegaly was detected on chromosome 16 (*Lmr18*) (**Figure 1C**), whereas on chromosome 4 (*Lmr24*), only linkage to IFNγ level reached the significance threshold (**Figure 1D**).

### Analysis of interval-specific congenic strains confirmed the linkage to *Lmr15* and revealed the presence of at least two functionally distinct loci

**Figure 2:**
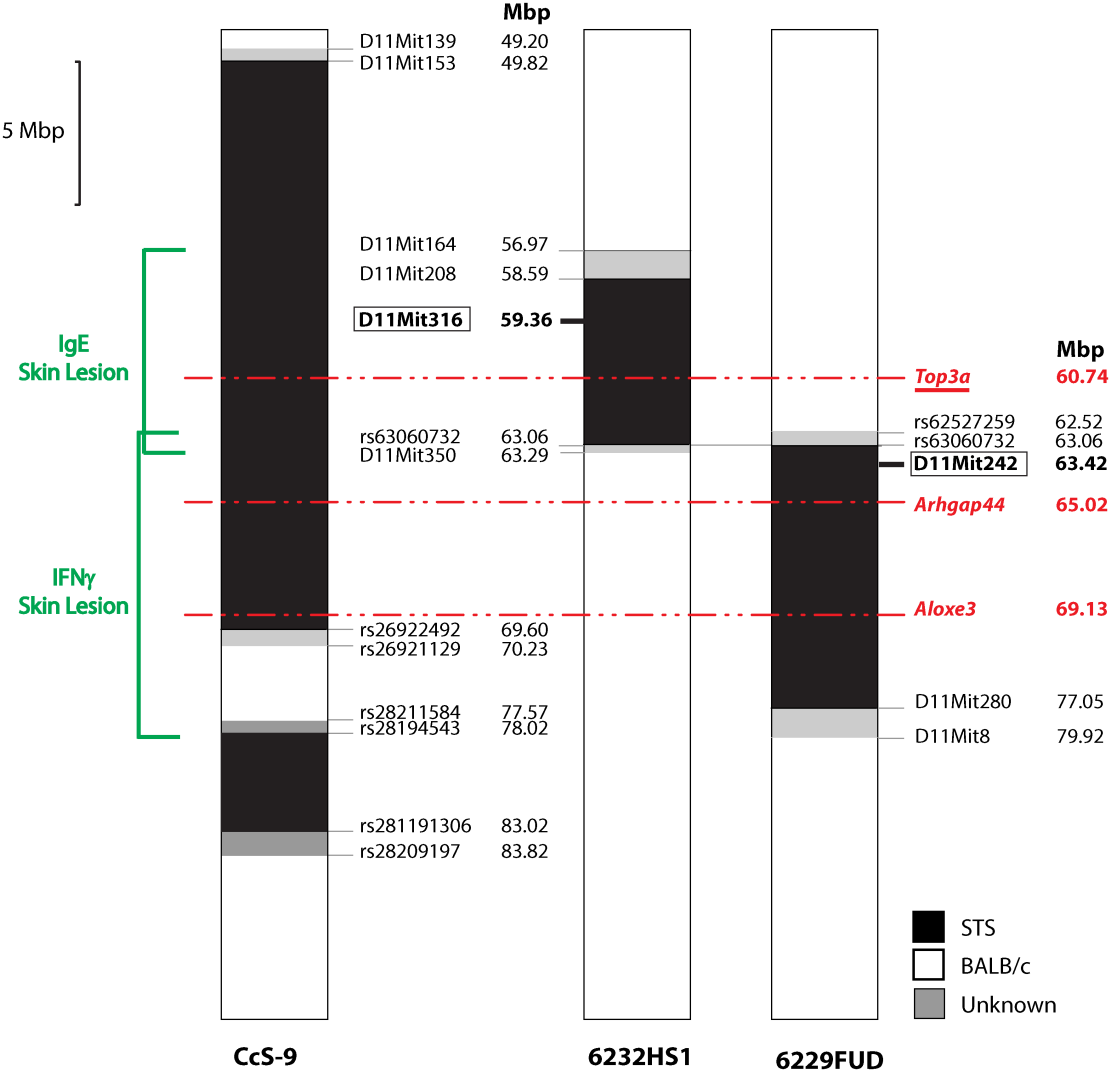
Maps of recombinants in *Lmr15* on chromosome 11. The regions of STS and BALB/c are represented as black and white, respectively; the boundary regions of undetermined origins are shaded. Red—potential candidate gene. Red, underlined—gene with alleles exhibiting differential expression.

Locus *Lmr15* on chromosome 11 was selected for further analysis because the linkage to this locus has been robust and because its control of the phenotype was stable. We have prepared two interval-specific congenic strains, 6232HS1 (HS1) and 6229FUD (FUD) (**Figure 2**), that overlap in a short segment of 0.77 Mbp spanning from rs62527257 (62.52 Mbp) to D11Mit350 (63.29 Mbp).

**Figure 3:**
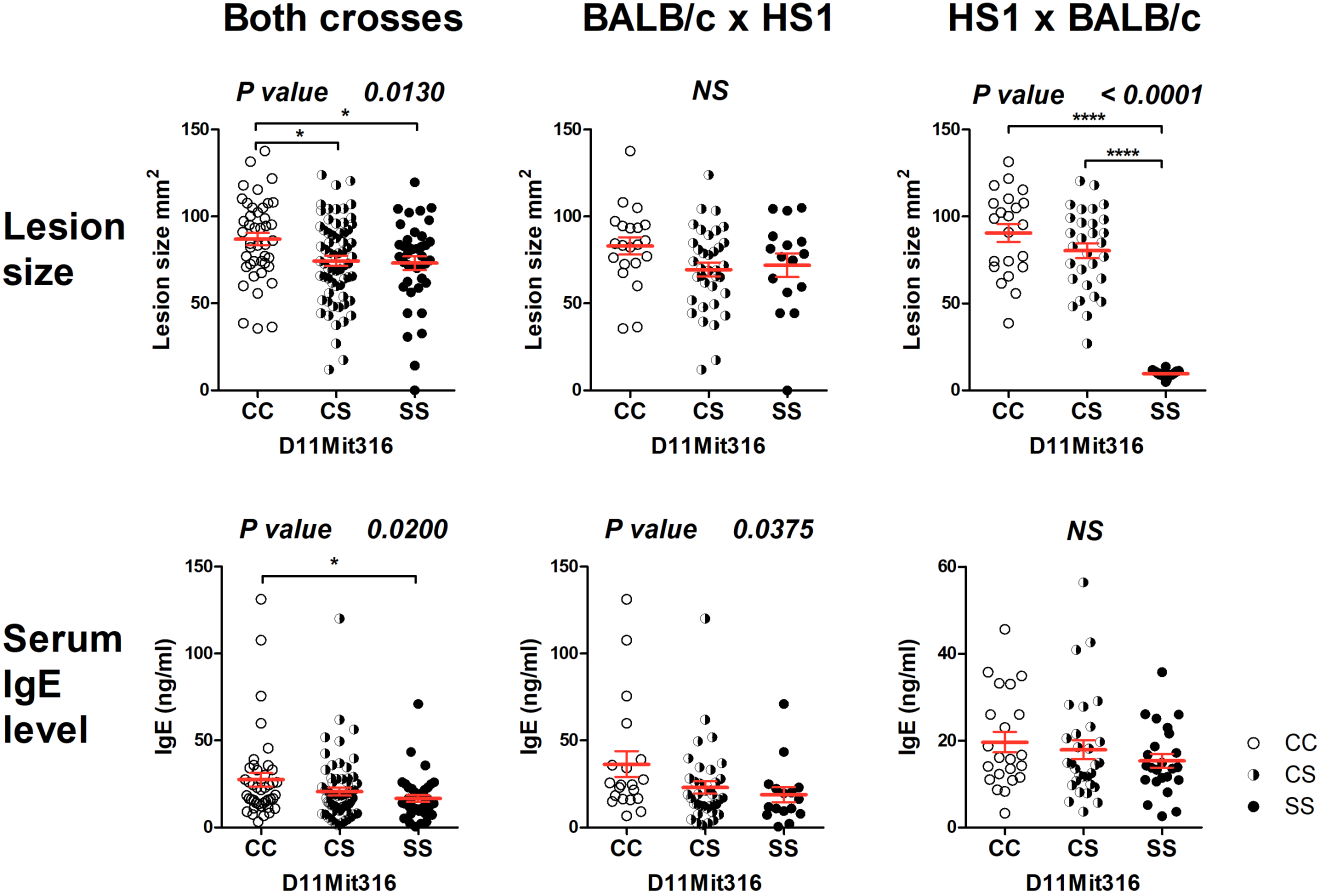
Genetic influence on skin lesions and serum IgE level 8 weeks after infection. Individual F_2_ hybrid mice between the strains HS1 and BALB/c are shown. Means ± standard error mean (red lines) and *P*-values were calculated by analysis of variance (ANOVA) followed by Bonferroni’s multiple comparison test. In order to obtain normal distribution of IgE values required for ANOVA, the absolute value of the logarithm of IgE (ng/ml) was used. Values of skin lesions had a normal distribution. The image shows untransformed values. C and S indicate the presence of the BALB/c and STS alleles, respectively. NS, not significant. *P < 0.05; ****P < 0.0001.

The strain HS1 carrying more proximal STS segment (maximal length 6.32 Mbp, minimal length 4.47 Mbp) controls skin lesion size (*P* = 0.013) and IgE level in the serum (*P* = 0.020) (**Figure 3**). The linkage to skin lesions was observed in the cross between HS1 females and BALB/c males (*P* < 0.0001) and in the group comprising both crosses (*P* = 0.013), and linkage to IgE was observed in the group comprising both crosses (*P* = 0.020) and in the cross between BALB/c females and HS1 males (*P* = 0.0375). Larger lesions and higher IgE levels are controlled by the BALB/c allele.

**Figure 4:**
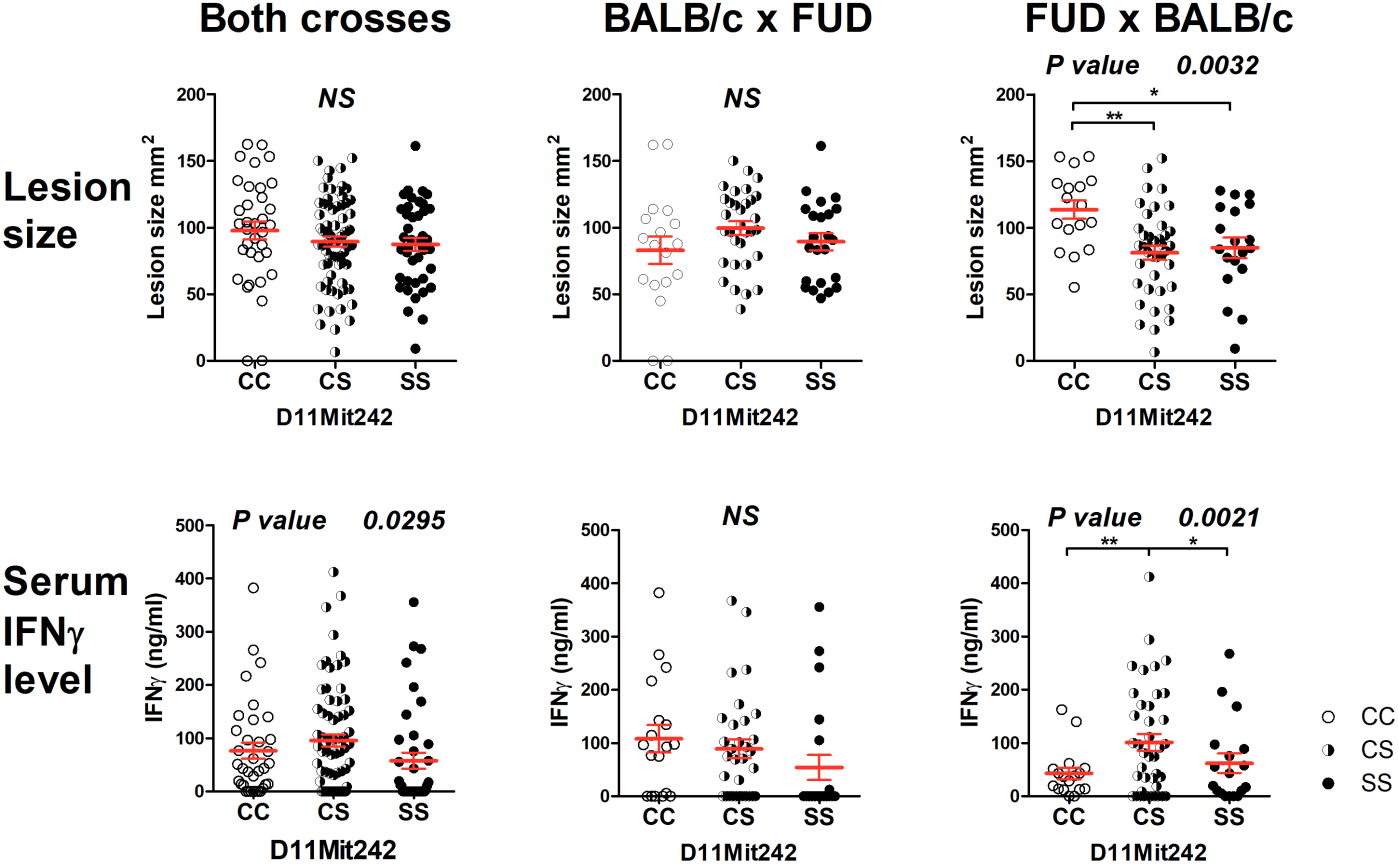
Genetic influence on skin lesions and serum IFNγ level 8 weeks after infection. Individual F_2_ hybrid mice between the strains FUD and BALB/c are shown. Means ± standard error mean (red lines) and *P*-values were calculated by analysis of variance (ANOVA) followed by Bonferroni’s multiple comparison test. In order to obtain normal distribution of IFNγ values required for ANOVA, the logarithm of IFNγ (ng/ml) was used. Values of skin lesions had normal distribution. The image shows untransformed values. C and S indicate the presence of the BALB/c and STS alleles, respectively. NS, not significant. *P < 0.05; **P 0.01.

The strain FUD carrying the more distal STS-derived segment (maximal length 17.40 Mbp, minimal length 13.99 Mbp) (**Figure 2**) controls skin lesion size (*P* = 0.0032) and IFNγ level (*P* = 0.0021) in the cross between FUD females and BALB/c males. FUD controls IFNγ level also in the group comprising both crosses (0.0295) (**Figure 4**). Larger lesions were controlled by the C allele, whereas the highest IFNγ levels were observed in heterozygotes.

*Lmr15* controls parasite load in the lymph nodes and liver in the cross between CcS-9 and BALB/c (29), but we did not observe linkage to parasite load in these organs neither in HS1 or FUD.

### Potential candidate genes

Bioinformatics analysis revealed three potential candidate genes (**Table 2**). One of these genes *Top3a* [topoisomerase (DNA) III alpha] is localized in the strain HS1, whereas the two other genes, *Arhgap44* (Rho GTPase activating protein 44) and *Aloxe3* (arachidonate lipoxygenase 3), are situated in the strain FUD (**Figure 2**).

**Table 2:** List of potential candidate genes controlling the response to *Leishmania major* in the interval-specific congenic strains HS1 and FUD.

Chr.	Position (bp)	Genotype reference C57BL/6	Genotype BALB/c	Genotype STS	Type of change	Protein position of AA	Reference AA	Alteration	Conservation score	Gene symbol	Gene name	Gene ID MGI	Gene ID NCBI
11	60,742,311	G/G	G/G	**A/A**	Single AA change	668	P	L	6	*Top3a*	Topoisomerase (DNA) III alpha	1197527	21975
11	65,023,188	G/G	G/G	**A/A**	Single AA change	427	S	F	9(S)	*Arhgap44*	Rho GTPase activating protein 44	2144423	216831
11	69,134,001	G/G	**A/A**	G/G	Single AA change	341	G	S	3	*Aloxe3*	Arachidonate lipoxygenase 3	1345140	23801

The conservation score is inferred from the ConSurf software on 24 September 2020. The conservation score ranging from 1 to 9 is followed in brackets by the type of the residue (S, structural). The higher the score, the more conserved the altered residue. Red under the column “Genotype” marks the difference from the reference genotype. AA, amino acid.

**Figure 5:**
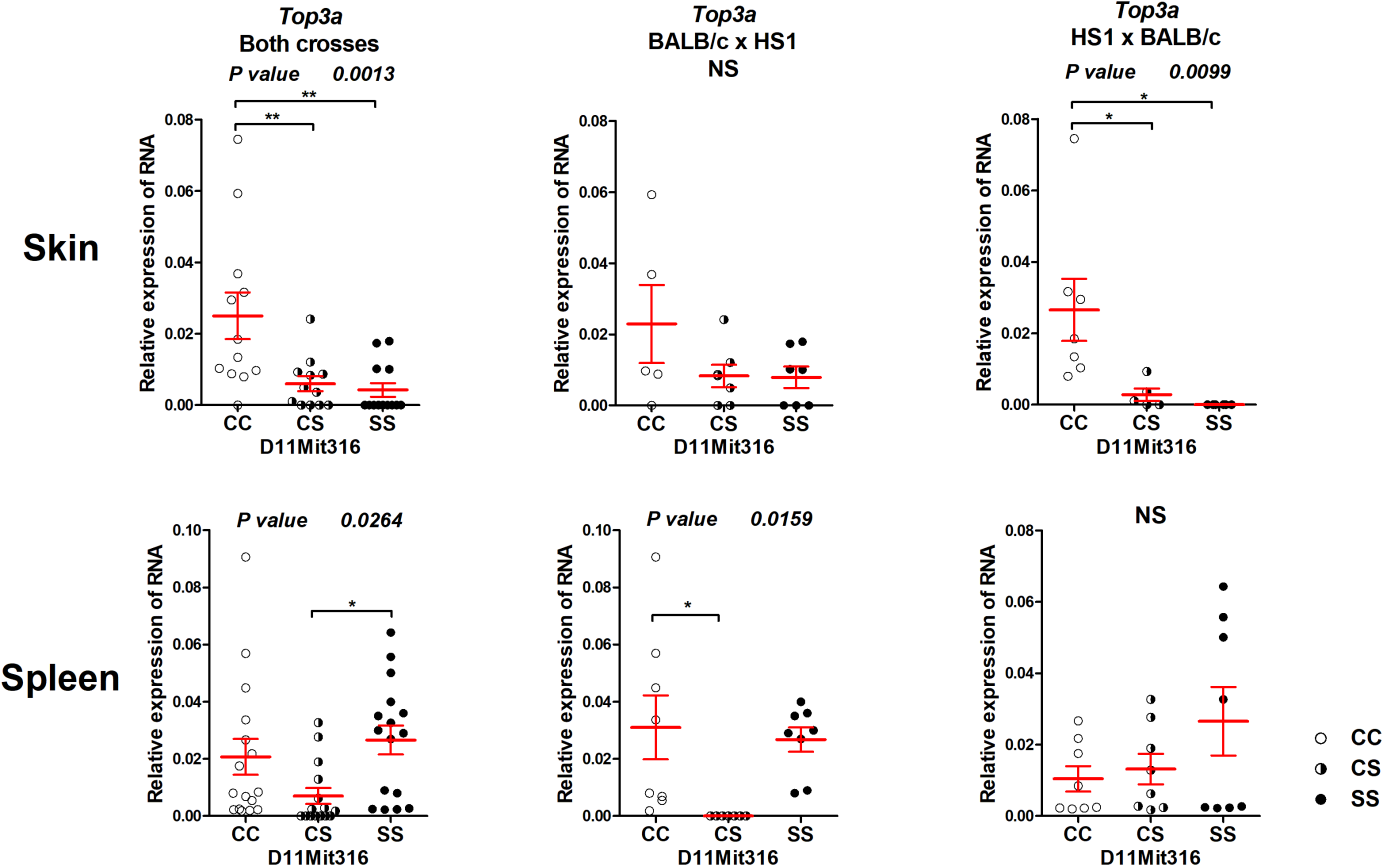
Expression of mRNA of the gene *Top3a* in the skins and spleens of F_2_ mice between HS1 and BALB/c 8 weeks after infection. Relative expression of a target gene *versus* the reference gene *Gapdh* is shown. C and S indicate the presence of the BALB/c and STS alleles, respectively. NS, not significant. Statistical analysis was performed by ANOVA followed by Bonferroni’s multiple comparison test. *P*-values are as indicated. Bars represent the average ± SEM. *P < 0.05; **P < 0.01.

The gene *Top3a* exhibited differential expression both in the skin and spleen (**Figure 5**). The CC homozygotes in the marker D11Mit316 exhibited the highest expression, whereas the SS homozygotes exhibited the lowest expression in both crosses and in the cross between HS1 females and BALB/c males, in which linkage to skin lesions was observed (**Figure 3**). In the spleen, differential expression was observed in both crosses and in the cross between BALB/c females and HS1 males. The lowest expression was observed in heterozygotes.

**Figure 6:**
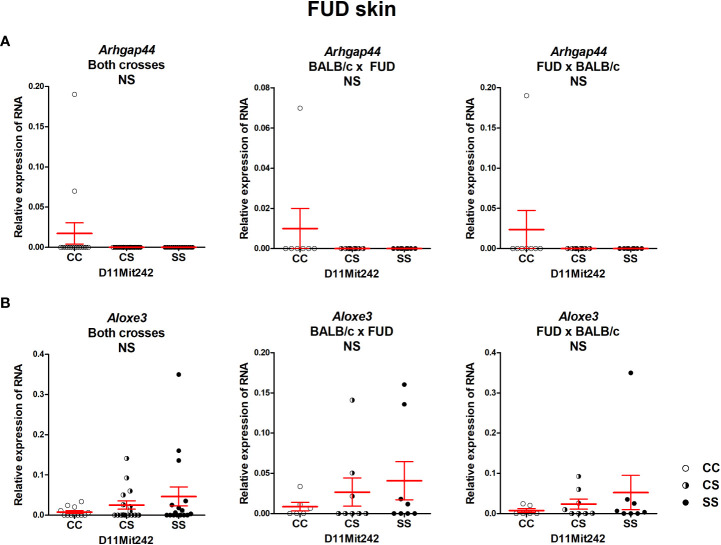
Expression of mRNA of the genes *Arhgap44* **(A)** and *Aloxe3* **(B)** in the skins of F_2_ mice between FUD and BALB/c 8 weeks after infection. Relative expression of a target gene *versus* the reference gene *Gapdh* is shown. C and S indicate the presence of the BALB/c and STS alleles, respectively. NS, not significant. Statistical analysis was performed by ANOVA followed by Bonferroni’s multiple comparison test. Bars represent the average ± SEM.

No differential expression of the genes *Arhgap44* and *Aloxe3*, situated in the segment FUD, was observed (**Figure 6**).

## Discussion

Mapping in AIL confirmed the linkages of the loci *Lmr15*, *Lmr27*, *Lmr24*, and *Lmr18* that were previously detected in F_2_ hybrids between BALB/c and CcS-9 (**Table 1**). We tested the linkage to skin lesions, splenomegaly, hepatomegaly, and IFNγ and IgE levels in the serum. In the next paragraphs, we will concentrate on the comparison of detection of the five phenotypes of the *Lmr15* locus in F_2_ hybrids and in AIL.

*Lmr15* was previously detected on chromosome 11 in two recombinant congenic strains: CcS-9 (17, 29) (maximal length 21.03 Mbp) and CcS-16 (26, 62) (maximal length 23.04 Mbp) (**Figure 1**). *Lmr15* detected in CcS-9 controls skin lesions, splenomegaly, hepatomegaly, and IL-4 and IgE in the serum, as well as parasite load in the lymph nodes and liver and eosinophil infiltration into the lymph nodes (17, 29). The STS-derived segment on chromosome 11 present in the strain CcS-16 (**Figure 1**) controls hepatomegaly (62) and IFNγ in the serum (26). Analysis of AIL K3FV also confirmed the position of *Lmr15*. AIL mapping detected on chromosome 11 the linkage to skin lesion size and IgE level in the serum and a weak linkage to splenomegaly, hepatomegaly, and IFNγ. Thus, this linkage is robust and operates across different genetic backgrounds. Partial differences between linkages to phenotypes detected in F_2_ mapping and in AIL described in this paper might be caused by differences in undetected gene interactions present in F_2_ hybrids and in AIL.

### *Top3a* is a potential candidate gene controlling skin lesions and IgE level in the segment HS1

The segment HS1 controls skin lesions in pooled crosses and in the cross HS1 × BALB/c (**Figure 3**). Significant differences in the expression of the potential candidate gene *Top3a* were also observed in pooled crosses and in the cross HS1 × BALB/c (**Figure 5**) and are similar to the differences observed in skin lesion size. *Top3a* is also differentially expressed in the spleen; the pattern of expression is different from those observed in the level of IgE in the serum.

TOP3A belongs to the eukaryotic type IA topoisomerases, TOP3A and TOP3B. Transcription and replication constantly change DNA topology, and topoisomerases are needed to relax supercoiling. TOP3A operates in both the nucleus and mitochondria and is involved in relaxing single-stranded DNA and RNA. TOP3A can couple its activity with different enzymes such as BLM (the Bloom syndrome DNA helicase) in dissolvasome, FANCM (Fanconi anemia group M protein) at replication forks, and PICH (an SNF2 family DNA translocase) during mitosis (63). Both FANCM (64) and BLM (65) are connected with the impairment of immune functions both in mouse and human. TOP3A was described to have a direct influence on T‐cell development in zebrafish (66). Thus, *Top3a* might be indirectly or directly involved in the immune response against leishmaniasis.

### None of the potential candidate genes in the segment FUD exhibited differential expression

The segment FUD controls skin lesions and serum IFNγ level. Bioinformatics analysis indicated two potential candidate genes: *Arhgap44* (Rho GTPase activating protein 44) and *Aloxe3* (arachidonate lipoxygenase 3). ARHGAP44 acts as a GTPase-activating protein (GAP) that stimulates the GTPase activity of Rho-type GTPases. It functions as a GAP for CDC42 (cell division cycle 42) and RAC1 (Rac family small GTPase 1) (67). CDC42 is involved in multiple cell functions including Th17 cell development (68) and regulation of neutrophil functions (69). ALOXE3 is expressed in the skin and belongs to 2-lipoxygenases that regulate tissue inflammation (70). Thus, both *Arhgap44* and *Aloxe3* have the potential to modify susceptibility to leishmaniasis, but none of them exhibited differential expression in the skin or spleen. We cannot exclude that they might influence susceptibility by the different activities of polymorphic proteins; however, the proof of this possibility is beyond the scope of this study.

### Overlap between HS1 and FUD unlikely controls any tested phenotype

A short overlap between HS1 and FUD (maximal length 0.77 Mbp) contains multiple regulatory elements (71). We did not detect any gene polymorphism that could influence gene functions and/or RNA stability. The distinct control of IgE and IFNγ levels by HS1 and FUD, respectively, implicated that these phenotypes are not controlled by this overlap. A comparison of the influence of C and S alleles on skin lesion size in HS1 and FUD seems to exclude the control of lesions by this segment. In HS1 (cross HS1 x BALB/c), the influence of the C allele is dominant (**Figure 3**), whereas in FUD (cross FUD x BALB/c), the S allele is dominant to the C allele (**Figure 4**).

### *Lmr15* and its co-localization with multiple disease-modifying QTLs

*Lmr15* overlaps with several loci involved in immune response, such as *Cinda1* (cytokine-induced activation 1) (72), *Tria1* (T-cell receptor-induced activation 1) (73), and *Mol4* (modifier of LPS-response 4) (74); loci that participate in response to malaria—*Char8* (*P. chabaudi* malaria resistance QTL 8) (75) and control the composition of the microbiome—*Micab14* (microbial abundance of Bacteroidales Bacteroidaceae Bacteroides 14) (76), and the susceptibility to autoimmunity comprising *Eae6* (experimental allergic encephalomyelitis susceptibility) (77), *Eae45* (experimental allergic encephalomyelitis susceptibility 45) (78), and *Acigg5* (anti-COL7 IgG2a/c antibody 5) (79). The question whether these loci are controlled by distinct or identical gene(s) could be answered after their identification.

### The strong influence of genetic background on the loci *Lmr18*, *Lmr24*, and *Lmr27*

AIL analysis of *Lmr15* confirmed both the linkages and phenotypes detected in F_2_ mapping, although linkages to some phenotypes did not reach the level of significance (**Figure 1A**; **Table 1**). The linkages to skin lesion size, IgE and IFNγ levels were further confirmed by recombinant mapping (**Figures 3**, **4**).

In AIL mapping of *Lmr18*, *Lmr24*, and *Lmr27*, the linkages to *L. major* response were confirmed, but these loci controlled the phenotypes that were different from those detected in F_2_ mapping (**Figures 1B–D**; **Table 1**).

Locus *Lmr15* and loci *Lmr18*, *Lmr24*, and *Lmr27* likely contain genes that are differently influenced by the genetic background. Similar variations in the alterations of gene effects by genetic background have been observed in other experimental designs and in human diseases. The underlying genetic basis is often unknown (80). In some cases, the phenotype of mice is entirely controlled by a mutation at the causative gene/locus, such as *Tyr* (tyrosinase); in others, for example, *Lep* (leptin), *Lepr* (leptin receptor), or *Fgfr2* (fibroblast growth factor receptor 2), this background has a dramatic effect on gene function. In more detail, the lack or mutation in *Tyr* invariantly leads to a white coat in mouse (81). On the other hand, the influence of *Fgfr2* on craniosynostosis is observed in C57BL/6, but not in BALB/c genetic background (82). Leptin-deficient BALB/cJ mice have a higher reduction in body weight and adiposity than leptin-deficient C57BL/6J mice, but they developed severe diabetes. C57BL/6J were sterile, whereas BALB/cJ were fertile (83). *Lepr* deficiency induces hyperglycemia and obesity in C57BL/6J mice but strong diabetes in the closely related strain C57BL/KsJ (84). Thus, a similar situation might take place in the interaction of *Lmr18*, *Lmr24*, and *Lmr27* with different genetic backgrounds.

### Newly detected locus on chromosome 17

AIL mapping detected the new locus *Lmr34* on chromosome 17 with a peak of linkage D17Mit139 (52.9 Mbp) that controls IgE level in the serum, skin lesion size, splenomegaly, and hepatomegaly (**Figure 1B**), which is probably distinct from *Lmr1* that spans from 10 to 86 Mbp and is linked to *H2* (35 Mbp) (24).

*Lmr34* encompasses several genes that participate in responses to *L. major* [CD70 (CD70 antigen)] (85) and to other *Leishmania* species [*SATB1* (special AT-rich sequence binding protein 1) (86) and *DPP9* (dipeptidylpeptidase 9) (87)] or genes that are components of pathways generally involved in response to *Leishmania* spp. {*Kat2b* [K(lysine) acetyltransferase 2B] (88), *Gtf2f1* [general transcription factor IIF, polypeptide 1] (89), *Ticam1* (TIR [Toll/IL-1 receptor] domain containing adaptor molecule 1) (90)}.

CD70 is a component of the IL-12-independent pathway, whereby a subset of dendritic cells induces IFNγ-secreting CD4^+^ T cells (85). *SATB1* is a gene with pleiotropic functions that include tissue repair. Patients suffering from cutaneous leishmaniasis with molecular evidence of persistence of *Leishmania* (*Viannia*) species in the nasal mucosa have a higher expression of *SATB1* in the nasal mucosa in comparison with patients with cutaneous leishmaniasis in which *Leishmania* was not detected (86). DPP9 represses the activation of the inflammasome NLRP1(NLR Family Pyrin Domain Containing 1) (87), which is involved in skin inflammation (91) and promotes susceptibility to experimental *L. braziliensis* infection (92). *Kat2b* [K(lysine) acetyltransferase 2B] participates in the epigenetic regulation of IL-10 (88). *Gtf2f1* (general transcription factor IIF, polypeptide 1) is involved in the pathway regulating CD4^+^ T-cell quiescence and exhaustion (89). *Ticam1* is a component of the TLR pathway that participates in the inflammatory response to *Leishmania* parasites (90).

*Lmr34* overlaps with QTLs controlling the response to other infectious diseases such as *Hbnr7* (*Heligmosomoides bakeri* nematode resistance 7) (peak 43–51.7 Mbp) (93), *Ari1* (antibody response to influenza 1, day 7, IgG2a+IgG2c) (peak 47.3–54.7 Mbp) (94), and *Plgr1* (plague resistance locus 1) (peak 48 Mbp) (95). This implies the presence either of clusters of functionally related genes or of gene(s) that participates in the control of several infections.

## Conclusion

The results indicate multidimensional analysis using RCS, AIL, interval-specific congenic strains, and bioinformatics tools as a novel approach in the fine mapping of genetic susceptibility of diseases.

We confirmed the previously detected loci *Lmr15*, *Lmr18*, *Lmr24*, and *Lmr27* and mapped one novel locus *Lmr34.* Genetic dissection of the effects of *Lmr15* on chromosome 11 revealed at least two linked but functionally distinct chromosomal regions controlling IFNγ response and IgE response, respectively, in addition to the control of skin lesions. Bioinformatics and expression studies led to the identification of the candidate gene *Top3a* that might influence resistance to leishmaniasis and, for the first time, highlighted the potential role of this gene in infection biology. We have also shown that the functional effects of the loci *Lmr18*, *Lmr24*, and *Lmr27* depend on genetic background. Thus, these experiments led to a better understanding of the genetic architecture of response to leishmaniasis, even if the mouse model is not completely transferable to human leishmaniasis.

## Data availability statement

The datasets presented in this study can be found in online repositories. The names of the repository/repositories and accession number(s) can be found below: Genebank (BankIt2665970 chr11_BALB/c_lmr15_seq1 OQ351010, BankIt2665970 chr11_BALB/c_lmr15_seq2 OQ351011, BankIt2666002 chr11_BALB/c_lmr15_seq3 OQ351012, BankIt2666002 chr11_BALB/c_lmr15_seq4 OQ351013, BankIt2666003 chr11_STS_lmr15_seq1 OQ351014, BankIt2666003 chr11_STS_lmr15_seq2 OQ351015, BankIt2666006 chr11_STS_lmr15_seq3 OQ351016, BankIt2666006 chr11_STS_lmr15_seq4 OQ351017).

## Ethics statement

The animal study was reviewed and approved by the Institutional Animal Care Committee of the Institute of Molecular Genetics and the Departmental Expert Committee for the Approval of Projects of Experiments on Animals of the Czech Academy of Sciences.

## Author contributions

IKr, YS and ML designed the project. IKr and ML wrote the manuscript. IKr, YS, HH, MČ, VV, IKu, JV and MS performed the experiments. IKr, YS, ESG, HS, VV, PD and ML analyzed the data. All authors contributed to the article and approved the submitted version.

## References

[1] Alvar J Vélez ID Bern C Herrero M Desjeux P Cano J. Leishmaniasis worldwide and global estimates of its incidence. PloS One (2012) 7(5):e35671. doi: 10.1371/journal.pone.0035671 22693548 PMC3365071

[2] Gradoni L. A brief introduction to leishmaniasis epidemiology. In: The leishmaniases: old neglected tropical diseases. Springer (2018). p. 1–13.

[3] World Health Organization. Leishmaniasis. Available at: https://www.who.int/en/news-room/fact-sheets/detail/leishmaniasis (Accessed April 22, 2022).

[4] Kobets T Grekov I Lipoldova M. Leishmaniasis: prevention, parasite detection and treatment. Curr Med Chem (2012) 19(10):1443–74. doi: 10.2174/092986712799828300 22360481

[5] Kaye PM Mohan S Mantel C Malhame M Revill P Le Rutte E. Overcoming roadblocks in the development of vaccines for leishmaniasis. Expert Rev Vaccines (2021) 20(11):1419–30. doi: 10.1080/14760584.2021.1990043 PMC9844205 34727814

[6] Bogdan C Donhauser N Döring R Röllinghoff M Diefenbach A Rittig MG. Fibroblasts as host cells in latent leishmaniosis. J Exp Med (2000) 191(12):2121–30. doi: 10.1084/jem.191.12.2121 PMC2193203 10859337

[7] Schwing A Pisani DF Pomares C Majoor A Lacas-Gervais S Jager J. Identification of adipocytes as target cells for *Leishmania infantum* parasites. Sci Rep (2021) 11(1):21275. doi: 10.1038/s41598-021-00443-y 34711872 PMC8553825

[8] Lipoldová M Demant P. Genetic susceptibility to infectious disease: lessons from mouse models of leishmaniasis. Nat Rev Genet (2006) 7:294–305. doi: 10.1038/nrg1832 16543933

[9] Sakthianandeswaren A Foote SJ Handman E. The role of host genetics in leishmaniasis. Trends Parasitol (2009) 25(8):383–91. doi: 10.1016/j.pt.2009.05.004 19617002

[10] Oryan A Akbari M. Worldwide risk factors in leishmaniasis. Asian Pac J Trop Med (2016) 9(10):925–32. doi: 10.1016/j.apjtm.2016.06.021 27794384

[11] Burza S Croft SL Boelaert M. Leishmaniasis. Lancet (2018) 392(10151):951–70. doi: 10.1016/S0140-6736(18)31204-2 30126638

[12] Lipoldová M Demant P. Gene-specific sex effects on susceptibility to infectious diseases. Front Immunol (2021) 12:712688. doi: 10.3389/fimmu.2021.712688 34721380 PMC8553003

[13] Krayem I Lipoldová M. Role of host genetics and cytokines in *Leishmania* infection. Cytokine (2021) 147:155244. doi: 10.1016/j.cyto.2020.155244 33059974

[14] Lipoldová M Svobodová M Havelková H Krulová M Badalová J Nohýnková E. Mouse genetic model for clinical and immunological heterogeneity of leishmaniasis. Immunogenetics (2002) 54(3):174–83. doi: 10.1007/s00251-002-0439-7 12073146

[15] Babay BE Louzir H Kebaïer C Boubaker S Dellagi K Cazenave PA. Inbred strains derived from feral mice reveal new pathogenic mechanisms of experimental leishmaniasis due to *Leishmania major* . Infect Immun (2004) 72(8):4603–11. doi: 10.1128/IAI.72.8.4603-4611.2004 PMC470675 15271920

[16] Kobets T Havelková H Grekov I Volkova V Vojtíšková J Slapničková M. Genetics of host response to *Leishmania tropica* in mice - different control of skin pathology, chemokine reaction, and invasion into spleen and liver. PloS Negl Trop Dis (2012) 6(6):e1667. doi: 10.1371/journal.pntd.0001667 22679519 PMC3367980

[17] Slapničková M Volkova V Čepičková M Kobets T Šíma M Svobodová M. Gene-specific sex effects on eosinophil infiltration in leishmaniasis. Biol Sex Differ (2016) 7:59. doi: 10.1186/s13293-016-0117-3 27895891 PMC5120444

[18] Sacks D Noben-Trauth N. The immunology of susceptibility and resistance to *Leishmania major* in mice. Nat Rev Immunol (2002) 2(11):845–58. doi: 10.1038/nri933 12415308

[19] Rostamian M Niknam HM. (2019) *Leishmania tropica*: what we know from its experimental models. Adv Parasitol 104:1–38. doi: 10.1016/bs.apar.2018.11.001 31030767

[20] Harrington V Gurung P. Reconciling protective and pathogenic roles of the NLRP3 inflammasome in leishmaniasis. Immunol Rev (2020) 297(1):53–66. doi: 10.1111/imr.12886 32564424 PMC7643606

[21] Volpedo G Pacheco-Fernandez T Bhattacharya P Oljuskin T Dey R Gannavaram S. Determinants of innate immunity in visceral leishmaniasis and their implication in vaccine development. Front Immunol (2021) 12:748325. doi: 10.3389/fimmu.2021.748325 34712235 PMC8546207

[22] Lipoldová M Sohrabi Y. Role of interferon-induced GTPases in leishmaniasis. PloS Negl Trop Dis (2022) 16(1):e0010093. doi: 10.1371/journal.pntd.0010093 35085246 PMC8794175

[23] Beebe AM Mauze S Schork NJ Coffman RL. Serial backcross mapping of multiple loci associated with resistance to *Leishmania major* in mice. Immunity (1997) 6(5):551–7. doi: 10.1016/s1074-7613(00)80343-x 9175833

[24] Roberts LJ Baldwin TM Curtis JM Handman E Foote SJ. Resistance to *Leishmania major* is linked to the H2 region on chromosome 17 and to chromosome 9. J Exp Med (1997) 185(9):1705–10. doi: 10.1084/jem.185.9.1705 PMC2196292 9151907

[25] Lipoldová M Svobodová M Krulová M Havelková H Badalová J Nohýnková E. Susceptibility to *Leishmania major* infection in mice: multiple loci and heterogeneity of immunopathological phenotypes. Genes Immun (2000) 1(3):200–6. doi: 10.1038/sj.gene.6363660 11196712

[26] Havelková H Badalová J Svobodová M Vojtísková J Kurey I Vladimirov V. Genetics of susceptibility to leishmaniasis in mice: four novel loci and functional heterogeneity of gene effects. Genes Immun (2006) 7(3):220–33. doi: 10.1038/sj.gene.6364290 16511555

[27] Kosarová M Havelková H Krulová M Demant P Lipoldová M. The production of two Th2 cytokines, interleukin-4 and interleukin-10, is controlled independently by locus *Cypr1* and by loci *Cypr2* and *Cypr3*, respectively. Immunogenetics (1999) 49(2):134–41. doi: 10.1007/s002510050472 9887350

[28] Sakthianandeswaren A Curtis JM Elso C Kumar B Baldwin TM Lopaticki S. Fine mapping of *Leishmania major* susceptibility locus *lmr2* and evidence of a role for *Fli1* in disease and wound healing. Infect Immun (2010) 78(6):2734–44. doi: 10.1128/IAI.00126-10 PMC2876540 20368343

[29] Kobets T Čepičková M Volkova V Sohrabi Y Havelková H Svobodová M. Novel loci controlling parasite load in organs of mice infected with *Leishmania major*, their interactions and sex influence. Front Immunol (2019) 10:1083. doi: 10.3389/fimmu.2019.01083 31231359 PMC6566641

[30] Available at: http://www.informatics.jax.org/inbred_strains/mouse/docs/STS.shtml (Accessed April 23, 2023).

[31] Demant P Lipoldova M Svobodova M. Resistance to *Leishmania major* in mice. Science (1996) 274(5291):1392a. doi: 10.1126/science.274.5291.1392a 17772041

[32] Palus M Vojtíšková J Salát J Kopecký J Grubhoffer L Lipoldová M. Mice with different susceptibility to tick-borne encephalitis virus infection show selective neutralizing antibody response and inflammatory reaction in the central nervous system. J Neuroinflamm (2013) 10:77. doi: 10.1186/1742-2094-10-77 PMC3700758 23805778

[33] van der Gugten AA Röpcke G van Nie R Hilgers J. Mouse strain (STS/A) resistant to mammary tumor induction by hypophysial isografts. Cancer Res (1985) 45(8):3448–53. 4016729

[34] Moen CJ van der Valk MA Snoek M van Zutphen BF von Deimling O Hart AA. The recombinant congenic strains–a novel genetic tool applied to the study of colon tumor development in the mouse. Mamm Genome (1991) 1(4):217–27. doi: 10.1007/BF00352328 1686571

[35] Mori N Okumoto M van der Valk MA Imai S Haga S Esaki K. Genetic dissection of susceptibility to radiation-induced apoptosis of thymocytes and mapping of *Rapop1*, a novel susceptibility gene. Genomics (1995) 25(3):609–14. doi: 10.1016/0888-7543(95)80001-3 7759093

[36] Mori N van Wezel T van der Valk M Yamate J Sakuma S Okumoto M. Genetics of susceptibility to radiation-induced apoptosis in colon: two loci on chromosomes 9 and 16. Mamm Genome (1998) 9(5):377–80. doi: 10.1007/s003359900773 9545495

[37] Lipoldová M Kosarová M Zajícová A Holán V Hart AA Krulová M. Separation of multiple genes controlling the T-cell proliferative response to IL-2 and anti-CD3 using recombinant congenic strains. Immunogenetics (1995) 41(5):301–11. doi: 10.1007/BF00172155 7721352

[38] Holán V Lipoldová M Demant P. Identical genetic control of MLC reactivity to different MHC incompatibilities, independent of production of and response to IL-2. Immunogenetics (1996) 44(1):27–35. doi: 10.1007/BF02602654 8613140

[39] Markel P Shu P Ebeling C Carlson GA Nagle DL Smutko JS. Theoretical and empirical issues for marker-assisted breeding of congenic mouse strains. Nat Genet (1997) 17(3):280–4. doi: 10.1038/ng1197-280 9354790

[40] Laird PW Zijderveld A Linders K Rudnicki MA Jaenisch R Berns A. Simplified mammalian DNA isolation procedure. Nucleic Acids Res (1991) 19:4293. doi: 10.1093/nar/19.15.4293 1870982 PMC328579

[41] Šíma M Havelková H Quan L Svobodová M Jarošíková T Vojtíšková J. Genetic control of resistance to *Trypanosoma brucei brucei* infection in mice. PloS Negl Trop Dis (2011) 5(6):e1173. doi: 10.1371/journal.pntd.0001173 21666791 PMC3110168

[42] Sohrabi Y Havelková H Kobets T Šíma M Volkova V Grekov I. Mapping the genes for susceptibility and response to *Leishmania tropica* in mouse. PloS Negl Trop Dis (2013) 7(7):e2282. doi: 10.1371/journal.pntd.0002282 23875032 PMC3708836

[43] Grekov I Svobodová M Nohýnková E Lipoldová M. Preparation of highly infective *Leishmania* promastigotes by cultivation on SNB-9 biphasic medium. J Microbiol Methods (2011) 87(3):273–7. doi: 10.1016/j.mimet.2011.08.012 21889549

[44] Sádlová J Svobodová M Volf P. *Leishmania major*: effect of repeated passages through sandfly vectors or murine hosts. Ann Trop Med Parasitol (1999) 93(6):599–611. doi: 10.1080/0003498995810 10707105

[45] Kobets T Badalová J Grekov I Havelková H Svobodová M Lipoldová M. *Leishmania* parasite detection and quantification using PCR-ELISA. Nat Protoc (2010) 5:1074–80. doi: 10.1038/nprot.2010.68 20539283

[46] Kurey I Kobets T Havelková H Slapničková M Quan L Trtková K. Distinct genetic control of parasite elimination, dissemination, and disease after *Leishmania major* infection. Immunogenetics (2009) 61:619–33. doi: 10.1007/s00251-009-0392-9 PMC2744819 19705113

[47] Sohrabi Y Volkova V Kobets T Havelková H Krayem I Slapničková M. Genetic regulation of guanylate-binding proteins 2b and 5 during leishmaniasis in mice. Front Immunol (2018) 9:130. doi: 10.3389/fimmu.2018.00130 29467757 PMC5808352

[48] Arvidsson S Kwasniewski M Riaño-Pachón DM Mueller-Roeber B. QuantPrime–a flexible tool for reliable high-throughput primer design for quantitative PCR. BMC Bioinf (2008) 9:465. doi: 10.1186/1471-2105-9-465 PMC2612009 18976492

[49] Palus M Sohrabi Y Broman KW Strnad H Šíma M Růžek D. A novel locus on mouse chromosome 7 that influences survival after infection with tick-borne encephalitis virus. BMC Neurosci (2018) 19(1):39. doi: 10.1186/s12868-018-0438-8 29976152 PMC6034256

[50] Krayem I Sohrabi Y Javorková E Volkova V Strnad H Havelková H. Genetic influence on frequencies of myeloid-derived cell subpopulations in mouse. Front Immunol (2022) 12:760881. doi: 10.3389/fimmu.2021.760881 35154069 PMC8826059

[51] Bolger AM Lohse M Usadel B. Trimmomatic: a flexible trimmer for illumina sequence data. Bioinformatics (2014) 30(15):2114–20. doi: 10.1093/bioinformatics/btu170 PMC4103590 24695404

[52] Magoč T Salzberg SL. FLASH: fast length adjustment of short reads to improve genome assemblies. Bioinformatics (2011) 27(21):2957–63. doi: 10.1093/bioinformatics/btr507 PMC3198573 21903629

[53] Li H Durbin R. Fast and accurate long-read alignment with burrows-wheeler transform. Bioinformatics (2010) 26(5):589–95. doi: 10.1093/bioinformatics/btp698 PMC2828108 20080505

[54] McKenna A Hanna M Banks E Sivachenko A Cibulskis K Kernytsky A. The genome analysis toolkit: a MapReduce framework for analyzing next-generation DNA sequencing data. Genome Res (2010) 20(9):1297–303. doi: 10.1101/gr.107524.110 PMC2928508 20644199

[55] Robinson JT Thorvaldsdóttir H Winckler W Guttman M Lander ES Getz G. Integrative genomics viewer. Nat Biotechnol (2011) 29(1):24–6. doi: 10.1038/nbt.1754 PMC3346182 21221095

[56] Cingolani P Platts A Wang le L Coon M Nguyen T Wang L. A program for annotating and predicting the effects of single nucleotide polymorphisms, SnpEff: SNPs in the genome of *Drosophila melanogaster* strain w1118; iso-2; iso-3. Fly (Austin) (2012) 6(2):80–92. doi: 10.4161/fly.19695 22728672 PMC3679285

[57] Choi Y Chan AP. PROVEAN web server: a tool to predict the functional effect of amino acid substitutions and indels. Bioinformatics (2015) 31(16):2745–7. doi: 10.1093/bioinformatics/btv195 PMC4528627 25851949

[58] Ashkenazy H Erez E Martz E Pupko T Ben-Tal N. ConSurf 2010: calculating evolutionary conservation in sequence and structure of proteins and nucleic acids. Nucleic Acids Res (2010) 38(Web Server issue):W529–33. doi: 10.1093/nar/gkq399 PMC2896094 20478830

[59] Celniker G Nimrod G Ashkenazy H Glaser F Martz E Mayrose I. Et al, ConSurf: using evolutionary data to raise testable hypotheses about protein function. Israel J Chem (2013) 53(3-4):199–206. doi: 10.1002/ijch.201200096

[60] Ashkenazy H Abadi S Martz E Chay O Mayrose I Pupko T. ConSurf 2016: an improved methodology to estimate and visualize evolutionary conservation in macromolecules. Nucleic Acids Res (2016) 44(W1):W344–50. doi: 10.1093/nar/gkw408 PMC4987940 27166375

[61] Purcell S Neale B Todd-Brown K Thomas L Ferreira MAR Bender D. PLINK: a toolset for whole-genome association and population-based linkage analysis. Am J Hum Genet (2007) 81(3):559–75. doi: 10.1086/519795 PMC1950838 17701901

[62] Vladimirov V Badalová J Svobodová M Havelková H Hart AA Blazková H. Different genetic control of cutaneous and visceral disease after *Leishmania major* infection in mice. Infect Immun (2003) 71(4):2041–6. doi: 10.1128/IAI.71.4.2041-2046.2003 PMC152088 12654824

[63] Pommier Y Nussenzweig A Takeda S Austin C. Human topoisomerases and their roles in genome stability and organization. Nat Rev Mol Cell Biol (2022) 23(6):407–27. doi: 10.1038/s41580-022-00452-3 PMC8883456 35228717

[64] Fagerlie SR Bagby GC. Immune defects in fanconi anemia. Crit Rev Immunol (2006) 26(1):81–96. doi: 10.1615/critrevimmunol.v26.i1.40 16472069

[65] Cunniff C Bassetti JA Ellis NA. Bloom's syndrome: clinical spectrum, molecular pathogenesis, and cancer predisposition. Mol Syndromol (2017) 8(1):4–23. doi: 10.1159/000452082 28232778 PMC5260600

[66] Mönnich M Hess I Wiest W Bachrati C Hickson ID Schorpp M. Developing T lymphocytes are uniquely sensitive to a lack of topoisomerase III alpha. Eur J Immunol (2010) 40(9):2379–84. doi: 10.1002/eji.201040634 20623552

[67] Richnau N Aspenström P. Rich, a rho GTPase-activating protein domain-containing protein involved in signaling by Cdc42 and Rac1. J Biol Chem (2001) 276(37):35060–70. doi: 10.1074/jbc.M103540200 11431473

[68] Ladinsky MS Araujo LP Zhang X Veltri J Galan-Diez M Soualhi S. Endocytosis of commensal antigens by intestinal epithelial cells regulates mucosal T cell homeostasis. Science (2019) 363(6431):eaat4042. doi: 10.1126/science.aat4042 30846568 PMC6708280

[69] Tackenberg H Möller S Filippi MD Laskay T. The small GTPase Cdc42 is a major regulator of neutrophil effector functions. Front Immunol (2020) 11:1197. doi: 10.3389/fimmu.2020.01197 32595647 PMC7304460

[70] Kulkarni A Nadler JL Mirmira RG Casimiro I. Regulation of tissue inflammation by 12-lipoxygenases. Biomolecules (2021) 11(5):717. doi: 10.3390/biom11050717 34064822 PMC8150372

[71] Mouse genome informatics. Available at: http://www.mousephenotype.org (Accessed April 22, 2022).

[72] Krulová M Havelková H Kosarová M Holán V Hart AA Demant P. IL-2-induced proliferative response is controlled by loci *Cinda1* and *Cinda2* on mouse chromosomes 11 and 12: a distinct control of the response induced by different IL-2 concentrations. Genomics (1997) 42(1):11–5. doi: 10.1006/geno.1997.4694 9177770

[73] Havelková H Krulová M Kosarová M Holán V Hart AA Demant P. Genetic control of T-cell proliferative response in mice linked to chromosomes 11 and 15. Immunogenetics (1996) 44(6):475–7. doi: 10.1007/BF02602810 8824160

[74] Matesic LE De Maio A Reeves RH. Mapping lipopolysaccharide response loci in mice using recombinant inbred and congenic strains. Genomics (1999) 62(1):34–41. doi: 10.1006/geno.1999.5986 10585765

[75] Hernandez-Valladares M Rihet P ole-MoiYoi OK Iraqi FA. Mapping of a new quantitative trait locus for resistance to malaria in mice by a comparative mapping approach with human chromosome 5q31-q33. Immunogenetics (2004) 56(2):115–7. doi: 10.1007/s00251-004-0667-0 15118851

[76] Bubier JA Philip VM Quince C Campbell J Zhou Y Vishnivetskaya T. A microbe associated with sleep revealed by a novel systems genetic analysis of the microbiome in collaborative cross mice. Genetics (2020) 214(3):719–33. doi: 10.1534/genetics.119.303013 PMC7054020 31896565

[77] Baker D Rosenwasser OA O'Neill JK Turk JL. Genetic analysis of experimental allergic encephalomyelitis in mice. J Immunol (1995) 155(8):4046–51. doi: 10.4049/jimmunol.155.8.4046 7561115

[78] Karlsson J Zhao X Lonskaya I Neptin M Holmdahl R Andersson A. Novel quantitative trait loci controlling development of experimental autoimmune encephalomyelitis and proportion of lymphocyte subpopulations. J Immunol (2003) 170(2):1019–26. doi: 10.4049/jimmunol.170.2.1019 12517969

[79] Ludwig RJ Müller S Ad M Recke A Schmidt E Zillikens D. Identification of quantitative trait loci in experimental epidermolysis bullosa acquisita. J Invest Dermatol (2012) 132(5):1409–15. doi: 10.1038/jid.2011.466 22297639

[80] Hou J van Leeuwen J Andrews BJ Boone C. Genetic network complexity shapes background-dependent phenotypic expression. Trends Genet (2018) 34(8):578–86. doi: 10.1016/j.tig.2018.05.006 PMC6085889 29903533

[81] Shibahara S Okinaga S Tomita Y Takeda A Yamamoto H Sato M. A point mutation in the tyrosinase gene of BALB/c albino mouse causing the cysteine–-serine substitution at position 85. Eur J Biochem (1990) 189(2):455–61. doi: 10.1111/j.1432-1033.1990.tb15510.x 2110899

[82] Dudakovic A Nam HK Wijnen AJV Hatch NE. Genetic background dependent modifiers of craniosynostosis severity. J Struct Biol (2020) 212(3):107629. doi: 10.1016/j.jsb.2020.107629 32976998 PMC7885185

[83] Qiu J Ogus S Mounzih K Ewart-Toland A Chehab FF. Leptin-deficient mice backcrossed to the BALB/cJ genetic background have reduced adiposity, enhanced fertility, normal body temperature, and severe diabetes. Endocrinology (2001) 142(8):3421–5. doi: 10.1210/endo.142.8.8323 11459786

[84] Hummel KP Coleman DL Lane P. The influence of genetic background on expression of mutations at the diabetes locus in the mouse C57BL/KsJ and C57BL/6J strains. Biochem Genet (1972) 7(1):1–13. doi: 10.1007/BF00487005 4557514

[85] Soares H Waechter H Glaichenhaus N Mougneau E Yagita H Mizenina O. A subset of dendritic cells induces CD4+ T cells to produce IFN-gamma by an IL-12-independent but CD70-dependent mechanism in vivo. J Exp Med (2007) 204(5):1095–106. doi: 10.1084/jem.20070176 PMC2118574 17438065

[86] Gómez-Zafra MJ Navas A Jojoa J Murillo J González C Gómez MA. Immune profile of the nasal mucosa in patients with cutaneous leishmaniasis. Infect Immun (2020) 88(5):e00881–19. doi: 10.1128/IAI.00881-19 PMC7171237 32094254

[87] Hollingsworth LR Sharif H Griswold AR Fontana P Mintseris J Dagbay KB. DPP9 sequesters the c terminus of NLRP1 to repress inflammasome activation. Nature (2021) 592(7856):778–83. doi: 10.1038/s41586-021-03350-4 PMC8299537 33731932

[88] Zheng Z Huang G Gao T Huang T Zou M Zou Y. Epigenetic changes associated with interleukin-10. Front Immunol (2020) 11:1105. doi: 10.3389/fimmu.2020.01105 32582189 PMC7287023

[89] Marasca F Sinha S Vadalà R Polimeni B Ranzani V Paraboschi EM. LINE1 are spliced in non-canonical transcript variants to regulate T cell quiescence and exhaustion. Nat Genet (2022) 54(2):180–93. doi: 10.1038/s41588-021-00989-7 35039641

[90] Muxel SM Acuña SM Aoki JI Zampieri RA Floeter-Winter LM. Toll-like receptor and miRNA-let-7e expression alter the inflammatory response in *Leishmania amazonensis*-infected macrophages. Front Immunol (2018) 9:2792. doi: 10.3389/fimmu.2018.02792 30555476 PMC6283264

[91] Fenini G Karakaya T Hennig P Di Filippo M Beer HD. The NLRP1 inflammasome in human skin and beyond. Int J Mol Sci (2020) 21(13):4788. doi: 10.3390/ijms21134788 32640751 PMC7370280

[92] Gupta G Santana AKM Gomes CM Turatti A Milanezi CM Bueno Filho R. Inflammasome gene expression is associated with immunopathology in human localized cutaneous leishmaniasis. Cell Immunol (2019) 341:103920. doi: 10.1016/j.cellimm.2019.04.008 31078283

[93] Menge DM Behnke JM Lowe A Gibson JP Iraqi FA Baker RL. Mapping of chromosomal regions influencing immunological responses to gastrointestinal nematode infections in mice. Parasite Immunol (2003) 25(6):341–9. doi: 10.1046/j.1365-3024.2003.00640.x 14507332

[94] Noll KE Whitmore AC West A McCarthy MK Morrison CR Plante KS. Complex genetic architecture underlies regulation of influenza-a-virus-specific antibody responses in the collaborative cross. Cell Rep (2020) 31(4):107587. doi: 10.1016/j.celrep.2020.107587 32348764 PMC7195006

[95] Turner JK McAllister MM Xu JL Tapping RI. The resistance of BALB/cJ mice to *Yersinia pestis* maps to the major histocompatibility complex of chromosome 17. Infect Immun (2008) 76(9):4092–9. doi: 10.1128/IAI.00488-08 PMC2519398 18573896

